# Effect of Feeding Olive Pomace Acid Oil on Dark Chicken Meat Lipid Composition, Oxidative Stability, Color, and Sensory Acceptance

**DOI:** 10.3390/ani13081343

**Published:** 2023-04-13

**Authors:** Paula Albendea, Francesc Guardiola, Magdalena Rafecas, Stefania Vichi, Ana C. Barroeta, Marçal Verdú, Alba Tres

**Affiliations:** 1 Nutrition, Food Science and Gastronomy Departament-XIA, Facultat de Farmàcia i Ciències de l’Alimentació, Campus de l’Alimentació de Torribera, Universitat de Barcelona, Av. Prat de la Riba, E-08921 Santa Coloma de Gramenet, Spainatres@ub.edu (A.T.); 2 Institut de Recerca en Nutrició i Seguretat Alimentària (INSA-UB), Universitat de Barcelona, Av. Prat de la Riba, E-08921 Santa Coloma de Gramenet, Spain; 3 Nutrition, Food Science and Gastronomy Departament-XIA, Facultat de Farmàcia i Ciències de l’Alimentació, Campus Diagonal, Universitat de Barcelona, Av. de Joan XXIII, E-08028 Barcelona, Spain; 4 Animal Nutrition and Welfare Service (SNiBA), Animal and Food Science Department, Facultat de Veterinària, Universitat Autònoma de Barcelona, Edifici V, Travessera dels Turons, E-08193 Bellaterra, Spain; 5 Department of Animal Nutrition and Feed Industry, bonÀrea Agrupa, E-25210 Guissona, Spain

**Keywords:** edible oil refining by-product, upcycling, chicken feed, chicken diet, poultry meat quality, fat by-product, broiler meat, meat refrigeration, palm oil, olive pomace oil

## Abstract

**Simple Summary:**

One current aim of the chicken meat industry is to reduce production costs, which can be achieved using by-products as feed ingredients. Additionally, this can contribute to the food chain sustainability. This study evaluated lipid composition, lipid oxidation, and quality of fresh and refrigerated chicken meat when an edible oil refining by-product (olive pomace acid oil) was added as a fat source (6%) in broiler diets, using refined olive pomace oil and crude palm oil as fat controls. Results showed that the use of olive pomace acid oil resulted in a similar meat fatty acid composition to the use of refined olive pomace oil, in both cases being rich in oleic acid, and in lower meat α-tocopherol levels compared to the use of palm oil. It did not affect meat lipid oxidation, its color, or its overall acceptance even after its refrigerated storage under commercial conditions, as compared to both control fats. Refrigeration increased meat lipid oxidation, redness, and yellowness, but its overall acceptance was not affected. Thus, considering these results, the olive pomace acid oil used in this study is an adequate fat source for broiler feeds in the conditions used in this study.

**Abstract:**

This study evaluated the effect of using olive pomace acid oil (OPAO) instead of crude palm oil (PO) or refined olive pomace oil (ROPO) on lipid composition, lipid oxidation, and quality of chicken meat. Broiler chickens were fed diets with 6% of PO, ROPO, or OPAO, and deboned legs with skin were sampled. Fresh and refrigerated (commercial conditions; 7 days) chicken meat samples were assessed for fatty acid (FA) composition, tocopherol (T) and tocotrienol (T3) content, lipid oxidative stability, 2-thiobarbituric acid (TBA) values, volatile compounds, color, and sensory acceptance. Using ROPO and OPAO led to meat richer in monounsaturated FAs and OPAO to lower α-T levels compared to PO. Oxidative stability, TBA values, volatile compounds, and overall acceptance of meat were not affected by diet. Refrigeration increased TBA values and some volatile compounds’ concentrations, but it did not decrease redness or consumers’ overall acceptance. Therefore, the OPAO used was an adequate fat source for chicken diets at 6%, as it produced dark meat lower in saturated FAs than PO without affecting lipid oxidation or overall acceptance. According to this, upcycling OPAO as an energy source in chicken diets would be possible, which can contribute to the sustainability of the food chain.

## 1. Introduction

Poultry diets are commonly supplemented with fat sources to cover the energy and essential fatty acid (FA) requirements. There are a wide variety of fat sources that have been used in poultry nutrition, but currently, the fats most commonly used are crude oils of vegetable origin (e.g., soybean and palm oils). The selection of these fat sources is mostly driven by their cost, but an improvement in the environmental sustainability of poultry meat production might also be desirable. Consequently, fat by-products of the edible oil industry can be an attractive alternative to crude vegetable oils to supplement animal diets.

Olive oil production represents an important industry in the European Union, as this area is responsible for 68% of the world’s production (about 2 million tons per year) [1]. This industry also generates a significant amount of waste, which is approximately four times greater than the amount of olive oil produced [2]. The main by-product of the olive oil industry is olive pomace, which is a mixture of olive pulp and stone. Olive pomace is still rich in oil and characterized by its high concentration of phenolic compounds and other bioactive substances [3]. After removing the water from the olive pomace, the crude olive pomace oil is extracted and subjected to a refining process, usually chemical.

The chemical refining of crude oils, such as crude olive pomace oil, is performed to remove substances that are naturally present in their composition and are undesirable for the taste, stability, and appearance of the edible oil. These compounds might include, among others, particles, pigments, impurities, volatile compounds, and some contaminants. Likewise, oil refining aims to decrease the acidity of the final edible oil, which is achieved through the neutralization step in the chemical refining process. In the neutralization, an alkali treatment is used to remove free fatty acids (FFAs) in the form of soap stocks [4]. Acid oils (AOs), such as olive pomace acid oil (OPAO), are obtained as by-products after acidifying soap stocks. Therefore, AOs are characterized by a high content in FFAs, a high variability of composition, and an FA profile related to the botanical origin of the crude oil [4]. Therefore, OPAO is rich in oleic acid as other oils of olive origin and, as a by-product, represents an economical alternative to the conventional fat sources used in poultry nutrition. Moreover, the carbon footprint can be reduced by upcycling them in the food chain instead of using them in the chemical industry, as the latter always involves additional processing. Despite the benefits that the use of OPAO in animal nutrition may have, a few trials have been performed to evaluate its impact as feeding fat on growth parameters [5,6,7], whereas the information about its effect on the quality of animal products is scarcer [8]. Therefore, further research is needed to increase confidence in OPAO as a feed ingredient.

The objective of this study was to assess the impact of using OPAO as a fat source in chicken diets on the lipid composition, oxidative stability, color, and sensory acceptance of fresh and refrigerated dark chicken meat, in comparison to the effects of using crude palm oil (PO) and refined olive pomace oil (ROPO). This work complements the results obtained by Verge-Mèrida et al. [5] within the same study on growth and digestibility parameters.

## 2. Materials and Methods

### 2.1. Experimental Fat Sources

Three experimental fat sources were used: OPAO, provided by RIOSA (Refinación Industrial Oleícola S.A., Ibros, Spain); and PO and ROPO, provided by bonÀrea Agrupa (Guissona, Spain). The MIU value (sum of moisture and volatile matter (M), impurities (I) and unsaponifiable (U) fractions), the FA profile, the tocopherol (T) and tocotrienol (T3) contents, the lipid class composition, the acid value and the peroxide value of these fat sources were determined in triplicate as described in Varona et al. [9] (Table 1). In this study, ROPO was chosen as a control fat due to its similar FA profile to OPAO but with a much lower FFA content and MIU value, whereas PO was selected because of its common usage in broiler diets.

### 2.2. Animals and Diets

Chicken housing and husbandry were carried out at the animal experimental facilities of bonÀrea Agrupa (Nial farm, Guissona, Spain). All the procedures were performed according to the directive 2010/63/EU [10], and all management practices and procedures were approved by the Animal Ethics Committee (CEEAH) of the Universitat Autònoma de Barcelona (code number: 10167). A total of 3048 one-day-old newly hatched broiler chickens (Ross 308) were distributed into 24-floor pens (127 animals per pen), balanced by body weight (average of 40.83 ± 0.26 g). During the trial, the temperature and the relative humidity of the pens were controlled (average of 24.5 ± 1.7 °C and 73.5 ± 6.8%, respectively), and the animals had ad libitum access to feed and water.

The animals were fed with experimental diets (PO, ROPO, or OPAO diets) that consisted of a grower basal diet (from day 22 to day 29) or a finisher basal diet (from day 30 to day 39), supplemented with a 6% of one of the three fat sources of interest (PO, ROPO, or OPAO). The ingredient composition of grower and finisher diets is shown in Table 2. All diets were formulated to cover or exceed the nutritional requirements [11]. Diets were randomly assigned among the 24 pens, counting a total of 8 pens per diet.

Feed samples were collected and stored at −20 °C until analysis. The FA composition, T and T3 content, and the lipid fractions of feeds (Table 3) were determined following the methods described in the Supplementary Materials.

The effects of these diets on animal performance parameters have been published by Verge-Mèrida et al. [5], and no effect was observed on broiler body weight at 39 days of age.

### 2.3. Sampling of Dark Chicken Meat

After 17 days of feeding the experimental diets, broilers were fasted for 4 h and slaughtered following the habitual commercial procedure at the slaughterhouse of bonÀrea Agrupa (Guissona, Spain). To perform the sampling, each pen was considered as an experimental unit (24 experimental units = 3 diets × 8 pens per diet), and four female chickens from each pen were chosen. From each animal, deboned legs (thigh plus drumstick) with skin were taken as samples. One deboned leg per animal was used for the fresh dark meat study, and the other one for the refrigerated dark meat study. Thus, for each experimental unit, 4 legs were placed in a PET/PE tray for the fresh sample study, and the other 4 legs were placed in a different PET/PE tray for the refrigerated sample study. The trays were sealed with a PA/EVOH/PE film and kept under commercial conditions (O_2_/CO_2_; 70/30; 3–4 °C) until the following morning (fresh chicken meat) or for 7 days (refrigerated chicken meat).

For the fresh chicken meat study, the morning after the slaughter, the 4 legs of each tray were cut, pooled, and ground, and color determination was performed. Part of the ground sample was immediately vacuum-packed in various high-barrier multilayer bags (Cryovac BB3255; permeability to O_2_, 17 cm^3^/m^2^ per day per bar at 23 °C and 0% relative humidity, ASTMD-3985; Cryovac Europe, Sealed Air S. L., Sant Boi de Llobregat, Spain) and kept at −20 °C until the chemical parameters were determined (fresh dark meat with skin). The ground sample remains from the experimental units from the same diet (PO, OPO, or OPAO diet) were pooled and immediately used to prepare cooked burgers for the sensory acceptance test. Preparation of all fresh samples, including color determination and sensory acceptance testing, was carried out in one morning.

For the refrigerated storage study, the sampling process and color and sensory analysis were performed identically after 7 days of refrigeration.

### 2.4. Determination of Fatty Acid Composition

The FA composition was determined only in fresh dark chicken meat. The lipid fraction from 1 g of the sample was extracted with chloroform/methanol (2:1, *v*/*v*) [12]. The fatty acid methyl esters were obtained and determined by GC-FID, as described in Varona et al. [9]. Each FA was expressed in percentage after peak area normalization. The FA determination is described in more detail in Supplementary Materials.

### 2.5. Determination of Tocopherol and Tocotrienol Content

The fresh and refrigerated dark chicken meat was saponified, and the unsaponifiable compounds were extracted as described by Bou et al. [12], redissolving the final extract with an exact volume of n-hexane. Then, the content of Ts and T3s was determined by high-performance liquid chromatography with fluorescence detector (HPLC-FLD) under the chromatographic conditions described by Aleman et al. [13]. More details on the procedure can be found in Supplementary Materials.

### 2.6. Ferrous Oxidation-Xylenol Orange Assay

The ferrous oxidation orange xylenol (FOX) assay was carried out in fresh and refrigerated samples of dark chicken meat to evaluate the primary oxidation and oxidative stability [14]. After a methanolic extraction of lipid hydroperoxides (LHPs), the colorimetric reaction was performed as detailed by Tres et al. [15], but with 940 µL of methanol and 160 µL of sample extract for fresh samples, and with 950 µL of methanol and 150 µL of sample extract for refrigerated samples. Absorbance at 560 nm after an incubation of 30 min evaluated the LHPs present in the sample (named LHP content). After 96 h of incubation (when the absorbance was stable), the measurements revealed the amount of LHPs formed depending on meat susceptibility to oxidation (named final LHP value). A calibration curve with cumene hydroperoxide (CHP) standard was used to quantify the LHP concentrations (expressed as mmol of CHP equivalents/kg of the sample). More details on the procedure can be found in Supplementary Materials.

### 2.7. Determination of 2-Thiobarbituric Acid Value

The 2-thiobarbituric acid (TBA) value was measured for fresh and refrigerated samples of chicken meat as a secondary oxidation parameter. The determination was carried through third derivate spectrophotometry as described by Grau et al. [16], and the TBA value was expressed as malondialdehyde (MDA) concentration (µg/kg) [17]. For more detail, see Supplementary Materials.

### 2.8. Determination of Volatile Compound Content

The volatile compound content was analyzed in fresh and refrigerated dark chicken meat, according to Albendea et al. [8], by headspace solid-phase microextraction coupled with gas chromatography and mass spectrometry (HS-SPME-GC-MS). The quantification was performed in selected ion mode [8], and results were expressed as μg of 4-methyl-2-pentanol equivalents/kg of sample. For more detail, see Supplementary Materials.

### 2.9. Color Determination

Immediately after grinding the samples, the color of fresh and refrigerated dark chicken meat with skin was determined according to CIE L*a*b* color space [8].

To estimate if the differences in color parameters due to the refrigeration or the diet might be perceptible by the human eye, two dimensionless parameters ΔE were calculated:

- ΔE_R_: was calculated with Equation (1) to study the differences between the color of fresh and refrigerated chicken meat coming from each diet.

(1) $$\Delta E_{R}=\sqrt{{\left(L_{0d}^{*}-L_{7d}^{*}\right)}^{2}+{\left(a_{0d}^{*}-a_{7d}^{*}\right)}^{2}+{\left(b_{0d}^{*}-b_{7d}^{*}\right)}^{2}},$$

In Equation (1), $L_{0d}^{*}$, $a_{0d}^{*}$ and $b_{0d}^{*}$ were the L*, a* or b* means of fresh chicken meat from one of the diets (n = 8) and $L_{7d}^{*}$, $a_{7d}^{*}$ and $b_{7d}^{*}$ were the L*, a* or b* means of the refrigerated chicken meat from the same diet (n = 8).

- ΔE_D_: was obtained with Equation (2) to evaluate the differences between the color of refrigerated chicken meat coming from the diet with the edible oil refining by-product (OPAO) and refrigerated chicken meat from the other diets (ROPO or PO diets).

(2) $$\Delta E_{D}=\sqrt{{\left(L_{\mathrm{OPAO}}^{*}-L_{D}^{*}\right)}^{2}+{\left(a_{\mathrm{OPAO}}^{*}-a_{D}^{*}\right)}^{2}+{\left(b_{\mathrm{OPAO}}^{*}-b_{D}^{*}\right)}^{2}},$$

In Equation (2), $L_{\mathrm{OPAO}}^{*}$, $a_{\mathrm{OPAO}}^{*}$ and $b_{\mathrm{OPAO}}^{*}$ were the L*, a* or b* means of refrigerated chicken meat from the OPAO diet (n = 8) and $L_{D}^{*}$, $a_{D}^{*}$ and $b_{D}^{*}$ were the L*, a* or b* means of refrigerated chicken meat from ROPO or PO experimental diets (n = 8).

### 2.10. Sensory Acceptance Test

Two tests were carried out (one for fresh meat and the other one for refrigerated meat) to determine the overall acceptance of chicken meat, using a nine-point hedonic scale (1, “dislike extremely”; 5, “neither like nor dislike”; 9, “like extremely”). Hamburgers with 30 g of ground dark chicken meat with skin were prepared and cooked for 3.5 min, using a different machine for each diet. In each test, a total of 36 habitual consumers of chicken meat participated, each of whom evaluated one hamburger per dietary treatment.

### 2.11. Statistical Analysis

Multifactor ANOVA was carried out to evaluate the impact of the refrigeration time (0 and 7 days) and its interaction with the experimental diets (PO, ROPO, and OPAO) on the different parameters studied on fresh and refrigerated chicken meat. One-way ANOVA was performed to study the influence of the diets (PO, ROPO, and OPAO) on the FA profile of chicken meat (only for fresh samples); and on T and T3 composition, oxidation stability, TBA values, color, and sensory acceptance of fresh chicken meat or of refrigerated chicken meat. The differences among diets in fresh or refrigerated chicken meat were tested for multiple comparisons with Scheffé’s post hoc test. In all cases, differences were considered significant when *p* < 0.05. Statistic tests were carried out using the general linear model procedure of SPSS (27.0 version, IBM Statistics Inc. (Chicago, IL, USA)).

## 3. Results

### 3.1. Fatty Acid Profile of Dark Chicken Meat

The FA composition of fresh chicken meat from animals fed with three experimental diets is shown in Table 4 (see Table S3 in Supplementary Materials for a more detailed FA composition).

Compared with PO dark chicken meat, meat from ROPO and OPAO diets was richer in MUFAs (*p* < 0.001), particularly in oleic acid. The latter two meats also showed a lower proportion of palmitic acid and, consequently, of SFAs (*p* < 0.001). Regarding the PUFA fraction, there were differences in the percentages of linoleic acid (*p* = 0.046), n-6 PUFAs (*p* = 0.047), and total PUFAs (*p* = 0.039), but Scheffé’s post hoc test could not separate the means of the different diets. The highest linolenic acid and n-3 PUFA proportions were observed in OPAO meat and the lowest in PO meat (*p* < 0.001). Therefore, the use of the OPAO diet led to a FA profile in chicken meat very similar to the use of the ROPO diet.

### 3.2. Tocopherol and Tocotrienol Composition of Dark Chicken Meat

The T and T3 content found for fresh and refrigerated chicken meat from broiler chickens fed with the three diets of interest is presented in Table 5. Refrigeration only affected the levels of γ-T and α-T3.

Regarding the effect of the diet, fresh and refrigerated chicken meat coming from the PO diet showed higher α-T than OPAO meat (*p* = 0.013) and higher α-T3 levels than ROPO and OPAO meats (*p* < 0.001). The total T + T3 levels in fresh PO meat were higher than those in fresh OPAO meat (*p* = 0.007), whereas the PO diet led to the highest levels in refrigerated chicken meat (*p* < 0.001).

### 3.3. Lipid Hydroperoxide Content and Oxidative Stability of Dark Chicken Meat

The LHP content of fresh and refrigerated chicken meat was under the quantification limit, and therefore this data is not shown. The oxidative stability of chicken meat was not significantly affected by the refrigerated storage or the type of diet, showing in all cases final LHP values of ≈0.20 mmol of CHP/kg of sample.

### 3.4. 2-Thiobarbituric Acid Value of Dark Chicken Meat

**Figure 1:**
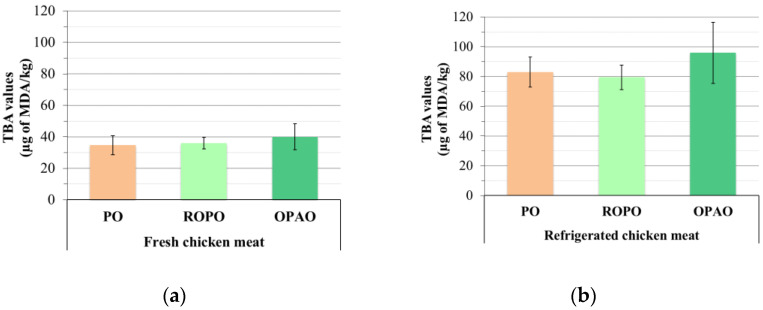
Thiobarbituric acid (TBA) values (µg of malondialdehyde (MDA)/kg) of (**a**) fresh and (**b**) refrigerated dark chicken meat with skin coming from the different diets (PO, crude palm oil; ROPO, refined olive pomace oil; OPAO, olive pomace acid oil). The results were represented as mean ± standard deviation (n = 8).

The TBA value of fresh and refrigerated chicken meat depending on the type of fat added to diets, is represented in Figure 1. Refrigeration increased (*p* < 0.001) the TBA value of chicken meat from 35–40 μg/kg to 79–96 μg/kg, whereas the type of experimental diet had no significant impact on either fresh or refrigerated meat.

### 3.5. Content of Volatile Compounds in Dark Chicken Meat

A total of eight volatile compounds were identified in fresh and refrigerated chicken meat (Table 6). Three of them were aldehydes (hexanal, 2-hexenal, and nonanal), and the other three were alcohols (1-pentanol, 1-hexanol, and 1-octen-3-ol). The 1-octen-3-one was the only ketone found in chicken meat. The other identified compound was 2-pentylfurane. The refrigerated storage of chicken meat increased the concentrations of hexanal (*p* = 0.002), 2-hexenal (*p* = 0.038), 1-pentanol (*p* < 0.001), 1-hexanol (*p* = 0.007) and 1-octen-3-ol (*p* < 0.001). The content of 1-octen-3-ol increased to a greater extent than the other volatile compounds. There was no effect of the diet on the volatile content of fresh or refrigerated chicken meat.

### 3.6. Color of Dark Chicken Meat

The values obtained for the three instrumental parameters of color (L*, a*, and b*) and for the two calculated ΔE parameters (ΔE_R_ and ΔE_D_) are presented in Table 7. There was a significant (*p* = 0.040) interaction between the refrigeration and the diet for meat yellowness (b*). Refrigeration increased meat yellowness for all diets (*p* < 0.001), but this increment was higher for ROPO chicken meat. Refrigeration also raised meat redness (*p* < 0.001). After refrigeration, there was an effect of the diet on meat lightness (*p* = 0.012), as ROPO refrigerated meat showed a higher lightness in comparison to PO refrigerated meat. Nevertheless, according to the dimensionless parameter ΔE_R_, overall, the color changes in chicken meat due to the refrigerated storage were similar for all the diets, finding values from 2.23 (PO meat) to 2.94 (ROPO meat). The ΔE_D_ obtained for refrigerated chicken meat showed that the color of OPAO meat was more similar to that of PO meat than to the color of ROPO meat.

### 3.7. Sensory Acceptance of Dark Chicken Meat

The sensory scores obtained for fresh and refrigerated chicken meat coming from the experimental diets are presented in Table 7. The sensory acceptance of chicken meat was not significantly affected by the refrigerated storage or the diet.

## 4. Discussion

### 4.1. Composition of Fat Sources and Diets

The three experimental fat sources used in this study presented differences in their MIU fraction, FA profile, T and T3 content, and lipid classes (Table 1). As reported by Varona et al. [4], the composition of some refining by-products, such as AOs, can be highly variable. In this study, OPAO showed the highest MIU value, as it had the highest M, I, and U contents. The MIU value of a fat represents a fraction of compounds that can dilute the energy provided by the fat. Thus, the energy dilution related to the MIU value was much higher in OPAO than in PO and ROPO. However, when OPAO was compared with olive AOs from the Spanish market, its MIU value (6.6 g/100 g) was close to the median value reported by Varona et al. [4] (7.6 g/100 g). Its FFA percentage (54.6%) and acid value (110.7 mg KOH/g) were the highest of the three fat sources, but they were in agreement with the range commonly observed for olive AOs (38.1–65.3% and 81.8–138.9 mg KOH/g, respectively) [4]. The peroxide value (7.0 meq O_2_/kg) showed by OPAO was lower than the value observed for ROPO (8.3 meq O_2_/kg), but it was higher than the median value obtained by Varona et al. [4] for similar AOs (1.3 meq O_2_/kg). However, all the fat sources used in this study met the peroxide value recommendations (<10 meq O_2_/kg) published in the national framework for fats with animal nutrition purposes [18].

Regarding the other compositional parameters, both the FA profile and the T and T3 composition of the three fat sources reflected their botanical origin. For instance, PO showed the highest SFA content and total T + T3 levels, being rich in T3s. With respect to OPAO, it showed similarities with ROPO, as both were rich in oleic acid, and their main tocol was α-T, which was in agreement with previous studies [4,19]. Despite these similarities between OPAO and ROPO due to the same botanical origin, the α-T content and total T + T3 level were lower in OPAO than in ROPO. Nevertheless, OPAO showed the highest U proportion (also compared to ROPO), which might indicate a higher content of some potentially bioactive compounds included in this fraction, such as squalene, some sterols or pigments, which are characteristic of olive pomace [3]. Overall, it can be assumed that the quality of the OPAO used in this study was similar to that of other AOs of olive origin in the Spanish market.

The FA composition of the diets was mainly influenced by the added fat source since it was added at 6%, and the total fat content of the diets was ≈8% (ether extract in Table 2). For instance, ROPO and OPAO diets had a similar FA profile and T and T3 composition. The other ≈2% of the total fat content of the diet came from the rest of the ingredients, mainly cereals. This explained the greater γ-T levels and PUFA proportions observed in the diets (Table 3) compared with the fat sources (Table 1), as some cereals are rich in Ts and T3s, especially in γ-T [20], and in linoleic acid [21,22]. However, the main contribution to the α-T and the total T + T3 levels in the diets was due to the premix, as it provided 100 mg of α-tocopheryl acetate/kg. Consequently, α-T was the main tocol in all diets, and the differences in the T + T3 levels observed between diets (Table 3) were attenuated in comparison to the differences found between the three fat sources (Table 1). Regarding the lipid class composition of the diets, the lipids provided by the rest of the ingredients were mainly TAGs, which caused the dilution of the FFAs provided by the OPAO (54.6%) in OPAO diets (29.8–30.7%).

### 4.2. Lipid Composition of Dark Chicken Meat

In this study, the FA composition of the diets was clearly mirrored by the FA profile of chicken meat. The fact that the FA profile of meat coming from monogastric animals can be easily modified with the diet has been widely reported in the literature [23,24,25,26]. In the present work, the main FA observed in dark chicken meat was oleic acid, and most of the FAs were MUFAs, even when the PO diet was used. This was consistent with Zaki et al. [27], who reported that chicken fed a diet containing 5% of PO produced thigh chicken meat with a higher proportion of MUFAs (40%) than SFAs (31%), with oleic acid being the main FA (33%). The use of OPAO in diets resulted in a FA composition of meat similar to the use of ROPO, with a higher MUFA content and a lower SFA content than when PO was used (*p* < 0.001). These results were in agreement with Crespo and Esteve-Garcia [28], as these authors also observed significantly higher MUFA (≈50%) and lower SFA (≈28%) proportions in thigh chicken meat when a 10% of olive oil was added to the diet than when a 10% of tallow was used (≈34% of SFAs; ≈41% MUFAs).

The differences in T and T3 concentrations found in fresh and refrigerated chicken meat (Table 5) reflected the different T and T3 levels observed in the finisher diets (Table 3). Even if in diets, the α-tocopheryl acetate supplementation attenuated the differences observed between fat sources (Table 1), the effect of the added fat source was still evident in meat, as OPAO meats showed a lower α-T content than PO meats (Table 5). Similarly, O’Neill et al. [29] also found differences in α-T concentrations in thigh meat from chickens fed with different fat sources (6% of olive oil or 6% of tallow), despite the fact that diets were supplemented with 200 mg/kg of α-tocopheryl acetate. The effect of the fat source was also noticeable in meat α-T3 content, as it was greater (*p* < 0.001) in PO meat. Thus, even if T3 uptake is not as efficient as that of α-T, dietary differences influenced meat T3 levels. This implication was also observed by Kang et al. [30], who found an increase in T3 levels (from 0.3 to 0.4 mg/kg) for hen white meat when the PO percentage added to the diet was raised from 1.5% to 3.5%.

### 4.3. Lipid Oxidation of Dark Chicken Meat

Despite primary oxidation (LHP content) being very low in fresh and refrigerated meat, secondary oxidation increased during the refrigerated storage under commercial conditions. According to the results of volatile compound composition and TBA value, the use of OPAO in chicken diets led to oxidation levels of fresh and refrigerated meat that were similar to those obtained with the use of ROPO or PO. Volatile compounds are responsible for the characteristic aroma and flavor of meat, but they can also cause a loss of its sensorial attributes. These compounds can be produced in meat by different pathways, such as lipid oxidation, amino acid degradation, or microbial action. For example, some authors have linked the formation of 1-octen-3-ol in chicken meat, which was the main volatile compound found in the present study, to the oxidation of linoleic acid or other PUFAs [31,32]. However, other authors related the formation of this alcohol to the presence of some bacteria [33,34]. Regarding the other aldehydes found in this study, hexanal might have been produced mainly due to the oxidation of linoleic and arachidonic acids, 2-hexenal due to linolenic acid oxidation, and nonanal due to n-9 MUFA oxidation [31,35]. The alcohols 1-pentanol and 1-hexanol, as well as 1-octen-3-one and 2-pentylfurane (Table 6), have also been associated with linoleic acid oxidation [31,36,37]. Therefore, all the volatile compounds identified in dark chicken meat in this study could mainly originate from lipid oxidation. Consequently, the increase in the content of hexanal, 2-hexenal, 1-pentanol, and, to a greater extent, 1-octen-3-ol with the refrigerated storage (Table 6) was consistent with the increase in the secondary oxidation products revealed by the TBA values (Figure 1). In the present study, the TBA values observed for fresh dark chicken meat with skin (35–40 μg/kg) were similar to the ones found by Botsoglou et al. [38] for dark chicken meat without skin (≈40 μg/kg). However, in refrigerated meat, the values (79–96 μg/kg) were lower than those observed by these authors in meat from the control diet after 6 days of storage wrapped in oxygen-permeable PVC stretch wrap at 4 °C (≈200 μg/kg). There are several reasons that may explain these differences, such as the more unsaturated diet (6% of soybean oil) used by these authors and the lower amount of α-tocopheryl acetate added to the control diet (30 mg/kg) compared to ours (100 mg/kg). In fact, the α-T levels that they achieved in fresh meat (3.5 mg/kg) were lower than those in our study (on average, 10.4 mg/kg). Moreover, the importance of using a properly modified atmosphere to prevent lipid oxidation in chicken meat has been previously reported by several authors [39,40,41].

Although the type of added fat (PO, ROPO, or OPAO) resulted in a different FA profile and total T + T3 levels in meat, it did not affect the oxidative parameters (LHP content and formation, TBA values, and volatile compound levels) neither in fresh nor in refrigerated meat. This outcome is in agreement with the results obtained by Narciso-Gaytán et al. [42], who reported similar TBA values for cooked chicken thighs coming from diets with 5% of a commercial blend of an animal–vegetable fat source, palm kernel oil, or soybean oil, all supplemented with 33 mg/kg of α-tocopheryl acetate. Oppositely, Grau et al. [43] found higher TBA values in fresh dark chicken meat with 46% of PUFAs than in meat with 18% of PUFAs that came from diets with 6% of linseed oil or 6% of beef tallow respectively. It is well known that the FA type with the highest tendency to suffer lipid oxidation are PUFAs, and this explains the differences in TBA values observed by Grau et al. [43]. In our study, the differences between the total PUFA proportions in chicken meat coming from the different diets (PO, ROPO, or OPAO) were minimal, as Scheffé’s post hoc test did not find differences between the diets used (21–23%). This contributes to explaining the similar behavior of chicken meat from the different diets in terms of lipid oxidation. Regarding the relation between lipid oxidation and α-T content, Lauridsen et al. [44] found lower TBA values in chicken thighs from a 10% olive oil diet than from a 10% tallow diet due to the higher α-T level provided by olive oil to the diet (57 mg/kg of α-T in the diet) compared with tallow (34 mg/kg of α-T in the diet). In the present study, the slightly lower α-T amount provided by the OPAO diets, even if it was reflected in the α-T levels of chicken meat, did not affect the lipid oxidation parameters.

### 4.4. Color of Dark Chicken Meat

The first perception of chicken meat quality by consumers is its color, so one important implication of this study is that the use of OPAO did not affect fresh meat color and that, after refrigeration, the color was still similar to the PO diet. The preservation of color during the refrigerated storage is an important goal for the meat industry. The most common effect of refrigeration on the color of chicken meat reported in the literature is its discoloration, which implies a decrease in redness [45,46]. This has been associated with the oxidation of myoglobin, which usually occurs together with lipid oxidation in chicken meat [47]. In the present study, refrigeration increased chicken meat redness (a*) (*p* < 0.001), showing a trend opposite to that related to the discoloration process. Therefore, color results obtained for chicken meat revealed that the modified atmosphere and the conditions of storage were adequate to avoid meat discoloration. Pogorzelska et al. [48] evaluated meat color differences by applying the National Bureau of Standards Unit (NBS unit) criteria that relate the ΔE parameter with differences between colors detectable by humans (0–0.5 trace; 0.5–1.5 slight; 1.5–3.0 noticeable; 3.0–6.0 appreciable; 6.0–12.0 much; >12.0 very much). According to this, the change in color due to refrigeration would be equally noticeable by the human eye (ΔE_R_ = 2.23–2.94) regardless of the diet used.

The color of chicken meat can also be influenced by pigments present in the diet, such as carotenoids, which can come from certain ingredients or from the use of commercial pigments [45]. Some of these liposoluble pigments can be deposited on chicken skin and intramuscular fat, enhancing the yellow color of fresh chicken meat. In this study, no color differences were observed between diets in fresh meat (Table 7), as the pigment contribution of the fat sources to the diets was minimum. This was due to the lower amount of fat source (6%) compared to other ingredients, such as corn, and due to the added commercial pigments (Table 2), and the type of pigments that they supplied. For instance, regarding the fat source, PO contains 500–700 mg/kg of carotenoids, the main ones being α- and β-carotene, which have a low pigmentation capacity [49], whereas oils from olives (ROPO and OPAO) are rich in chlorophyll pigments (generally in the range from 10 to 30 mg/kg) and have low content in carotenoids (usually less than 10 mg/kg, most of which are lutein and β-carotene) [50,51]. On the other hand, corn was one of the main ingredients (34–35%) used to formulate the diets, and it has around 10 mg/kg of lutein and 8 mg/kg of zeaxanthin [52]. Additionally, two different commercial pigments were added to the diets, with one of them being a natural extract of marigold flower (Indukern, El Prat del Llobregat, Spain). This pigment was added at 0.1 g/kg and had a total of 40 g/kg of carotenoids, of which 32 g/kg was lutein, and 2 g/kg was zeaxanthin, both of which provide a yellow color. The other commercial pigment was added at 0.05 g/kg and contained canthaxanthin at 10 g/kg (Industrial Tecnica Pecuaria, S.A., Barcelona, Spain), which intensifies the red color. Unlike fresh meat, diet significantly affected L* and b* in refrigerated meat, being higher for ROPO (Table 7). However, following the criteria applied by Pogorzelska et al. [48] to evaluate color differences between meats, the color difference between OPAO and PO refrigerated meats would only be slightly noticeable by the human eye (AE_D_ = 1.28) diets, whereas they would be noticeable between OPAO and ROPO diets (AE_D_ = 1.72).

### 4.5. Sensory Acceptance of Dark Chicken Meat

The results of this study showed that the use of OPAO at 6% in broiler diets did not affect the sensory acceptance of dark chicken meat, which is coherent with the lack of differences found between diets in the oxidative parameters. Many studies in the literature have focused attention on the relation between TBA values and sensory acceptance since the development of lipid oxidation in meat can cause oxidative rancidity and lead to a loss of meat sensory acceptance. For instance, Gray et al. [53] reported that an untrained taste panel detected off-odors and flavors in cooked meat when TBA values were in the range of 600–2000 μg MDA/kg. These authors also revealed that a trained taste panel detected oxidative rancidity when TBA values were between 500 and 1000 μg MDA/kg [53]. In this study, even if the TBA values (Figure 1) and the volatile compound levels (Table 6) significantly increased after the refrigeration, the sensory acceptance was not influenced by refrigeration or the diet used.

## 5. Conclusions

In conclusion, the addition of 6% of OPAO to broiler diets resulted in meat with a similar FA profile and T and T3 composition to the meat obtained with the use of 6% of ROPO. The use of both fat sources instead of PO led to meat richer in MUFAs and to some changes in the T and T3 profile. Additionally, the LHP content, the oxidative stability, the TBA values, the volatile compound concentrations, and the overall acceptance of fresh or refrigerated chicken meat were not affected by the fat source used in the diet. Moreover, the refrigeration conditions used in this study (O_2_/CO_2_; 70/30; 3–4 °C) were adequate to prevent the loss of redness and overall acceptance of meat from chicken fed either of the three fats (PO, ROPO, or OPAO). Thus, based on these findings, the OPAO used in this study is a suitable fat for broiler feed under the conditions used in this study. However, even if the OPAO used showed the typical quality found in the market, the composition of such by-products can vary significantly, and using them as feed ingredients may result in a varying effect on the quality of animal food products. Additionally, although different AOs may have a similar composition, the impact of using AOs in animal feed may differ depending on the species and age of the animals. Therefore, further studies are necessary to establish realistic recommendations regarding the composition that OPAO and other AOs should have to be adequate feed fats.

## Figures and Tables

**Table 1:** Characterization of the experimental fat sources.

	PO ^1^	ROPO ^1^	OPAO ^1^
MIU (g/100 g)			
Moisture ^2^	ND	ND	0.73 ± 0.01
Impurities	0.49 ± 0.03	0.28 ± 0.09	1.37 ± 0.21
Unsaponifiable	0.22 ± 0.11	1.44 ± 0.47	4.53 ± 0.30
Total	0.71 ± 0.12	1.72 ± 0.48	6.63 ± 0.37
FAs (%) ^3^			
C16:0	42.5 ± 0.05	12.7 ± 0.01	12.4 ± 0.14
C18:0	4.5 ± 0.01	2.7 ± 0.01	2.7 ± 0.89
SFAs	48.5 ± 0.03	16.2 ± 0.01	16.6 ± 0.74
C16:1 n-7	0.2 ± 0.01	0.9 ± 0.01	1.0 ± 0.01
C18:1 n-9	40.6 ± 0.05	70.0 ± 0.01	63.2 ± 0.61
C18:1 n-7	0.6 ± 0.01	1.6 ± 0.01	1.7 ± 0.01
MUFAs	41.6 ± 0.05	72.9 ± 0.02	66.2 ± 0.63
C18:2 n-6	9.5 ± 0.01	10.1 ± 0.02	15.8 ± 0.12
C18:3 n-3	0.3 ± 0.01	0.7 ± 0.01	0.9 ± 0.01
PUFAs	9.8 ± 0.01	10.8 ± 0.02	16.7 ± 0.13
Ts and T3s (mg/kg)			
α-T	178.40 ± 6.40	545.81 ± 4.28	266.16 ± 12.34
γ-T	2.29 ± 0.59	6.19 ± 0.49	15.25 ± 0.41
α-T3	215.24 ± 8.38	2.33 ± 1.08	2.08 ± 0.08
γ-T3	220.45 ± 2.95	ND	6.12 ± 0.73
T + T3	669.15 ± 16.63	579.68 ± 6.31	309.99 ± 12.47
Lipid classes (%) ^4^			
TAGs	88.0 ± 0.24	91.1 ± 0.09	24.5 ± 1.27
DAGs	8.7 ± 0.12	8.4 ± 0.14	18.6 ± 1.34
MAGs	0.1 ± 0.03	0.3 ± 0.01	2.3 ± 0.15
FFAs	3.31 ± 0.15	0.2 ± 0.04	54.6 ± 2.46
Acid value (mg KOH/g)	10.4 ± 0.07	0.3 ± 0.04	110.7 ± 0.26
Peroxide value (meq O_2_/kg)	3.8 ± 0.01	8.3 ± 0.11	7.0 ± 0.19

Abbreviations: PO, crude palm oil; ROPO, refined olive pomace oil; OPAO, olive pomace acid oil; MIU, moisture and volatile matter + insoluble impurities + unsaponifiable matter; FAs, fatty acids; SFAs, saturated fatty acids (sum of C14:0, C16:0, C18:0, C20:0, C22:0, and C24:0; see Table S1 in Supplementary Materials); MUFAs, monounsaturated fatty acids (sum of C16:1 n-7, C18:1 n-9, C18:1 n-7, and C20:1 n-9; see Table S1); PUFAs, polyunsaturated fatty acids (sum of C18:2 n-6, and C18:3 n-3); Ts, tocopherols; T3s, tocotrienols; T + T3, total tocol content (sum of α-T, β-T, γ-T, δ-T, α-T3, β-T3, γ-T3, and δ-T3); TAGs, triacylglycerols; DAGs, diacylglycerols; MAGs, monoacylglycerols; FFAs, free fatty acids; ND, not detected. ^1^ Data were expressed as mean ± standard deviation of three determinations. ^2^ It included moisture and other compounds that volatilize under the determination conditions. ^3^ The percentage of each FA was obtained by peak area normalization. See Table S1 for the complete FA composition, including minor FA results. ^4^ The percentage of each lipid class was obtained by peak area normalization.

**Table 2:** Ingredients, proximate composition, and gross energy of the grower and finisher experimental diets.

	Grower Diets ^1^	Finisher Diets ^1^
Ingredients (%)		
Corn	35.01	33.94
Wheat	10.85	15.01
Sorghum	10.00	10.00
Sunflower meal	10.00	10.00
Soybean meal 47%	23.79	19.74
Experimental fat ^2^	6.00	6.00
Calcium carbonate	0.69	1.02
Dicalcium phosphate	0.98	0.73
Sodium chloride	0.29	0.28
Silicate	-	1.00
Vitamin and mineral premix ^3^	0.67	0.67
DL-Methionine ^4^	0.28	0.24
L-Lysine ^5^	0.46	0.45
L-Threonine	0.09	0.08
L-Valine	0.01	0.01
Marigold flower extract ^6^	0.10	0.10
Canthaxanthin powder ^7^	0.05	0.05
Coccidiostat agent ^8^	0.60	0.60
Choline ^9^	0.04	0.03
Sodium hydrogencarbonate	0.07	0.07
Proximate composition (%) ^10^		
Dry matter	89.44	89.93
Crude protein	19.26	18.39
Ether extract	7.65	7.95
Crude fiber	4.64	4.70
Ash	5.34	5.66
Gross energy (kcal/kg)	4225.7	4226.0

^1^ Grower diets were used from 22 to 29 days and finisher diets from 30 to 39 days. ^2^ Crude palm oil (PO), refined olive pomace oil (ROPO), or olive pomace acid oil (OPAO). ^3^ Provides, per kg of feed: vitamin A, 1000 IU; vitamin D, 4700 IU; vitamin E, 100 mg (α-tocopheryl acetate); vitamin K, 4 mg; vitamin B1, 4 mg; vitamin B2, 8 mg; vitamin B6, 5 mg; vitamin B12, 0.02 mg; biotin, 0.3 mg; Cu, 13.1 mg (CuSO_4_); I, 1.3 mg (KI); Mn, 122 mg (MnO_2_); Se, 0.3 mg (Na_2_SeO_3_); Zn, 68 mg (ZnO); Fe, 142 mg (FeSO_4_); phytase, 1500 FYT; endo-β-1,4-xylanase, 24,000 BXU. ^4^ DL-2-hydroxy-4-methylthiobutanoic acid (HMTBa), the hydroxy analog of DL-methionine. ^5^ L-Lysine sulfate. ^6^ Contains a minimum of 40 g of carotenoids/kg, of which a minimum of 32 g/kg is *trans*-lutein, and 2 g/kg is *trans*-zeaxanthin. ^7^ Contains 10 g of canthaxanthin/kg. ^8^ Narasin at 10%. ^9^ Choline chloride 75% (*w*/*w*) aqueous solution. ^10^ Mean of values obtained for the three grower diets (PO, ROPO, and OPAO) and the three finisher diets (PO, ROPO, and OPAO), expressed on a fresh matter basis.

**Table 3:** Fatty acid, tocopherol and tocotrienol, and lipid class compositions of the grower and finisher experimental diets.

	Grower Diets			Finisher Diets		
	PO ^1^	ROPO ^1^	OPAO ^1^	PO ^1^	ROPO ^1^	OPAO ^1^
FAs (%) ^2^						
C16:0	31.7 ± 0.04	13.0 ± 0.04	14.9 ± 0.02	32.6 ± 0.20	13.6 ± 0.01	16.5 ± 0.65
C18:0	3.8 ± 0.01	2.7 ± 0.01	3.2 ± 0.01	3.8 ± 0.07	2.9 ± 0.01	3.2 ± 0.07
SFAs	37.6 ± 0.05	16.9 ± 0.05	19.7 ± 0.02	38.3 ± 0.12	17.6 ± 0.01	21.2 ± 0.81
C18:1 n-9	35.5 ± 0.04	54.7 ± 0.08	46.7 ± 0.11	35.9 ± 0.33	55.0 ± 0.01	47.8 ± 1.41
C18:1 n-7	1.1 ± 0.01	1.9 ± 0.03	1.7 ± 0.06	1.1 ± 0.02	1.8 ± 0.11	1.6 ± 0.08
MUFAs	36.9 ± 0.05	57.5 ± 0.04	49.3 ± 0.05	37.3 ± 0.32	57.3 ± 0.11	50.2 ± 1.51
C18:2 n-6	24.6 ± 0.02	24.4 ± 0.06	29.6 ± 0.03	23.6 ± 0.19	24.0 ± 0.10	27.3 ± 0.69
C18:3 n-3	0.9 ± 0.02	1.1 ± 0.02	1.3 ± 0.01	0.8 ± 0.01	1.0 ± 0.01	1.1 ± 0.01
PUFAs	25.5 ± 0.01	25.5 ± 0.09	30.8 ± 0.01	24.4 ± 0.20	25.0 ± 0.11	28.4 ± 0.70
Ts and T3s (mg/kg)						
α-T	99.46 ± 1.65	108.67 ± 2.65	96.09 ± 0.69	105.09 ± 1.00	102.64 ± 0.98	97.7 ± 1.14
γ-T	13.68 ± 0.03	13.12 ± 0.25	13.37 ± 0.02	17.63 ± 0.40	16.39 ± 1.52	17.46 ± 0.62
α-T3	13.98 ± 0.18	5.52 ± 0.11	5.87 ± 0.02	14.47 ± 0.37	3.48 ± 0.48	5.75 ± 0.414
γ-T3	18.35 ± 0.30	4.80 ± 0.06	6.11 ± 0.43	21.23 ± 0.32	3.52 ± 1.26	2.60 ± 0.41
T + T3	152.52 ± 2.22	138.36 ± 2.95	128.28 ± 0.28	168.06 ± 3.82	133.04 ± 4.65	130.93 ± 2.09
Lipid classes (%) ^3^						
TAGs	82.5 ± 0.21	84.1 ± 0.04	54.8 ± 0.20	84.2 ± 0.16	85.0 ± 0.16	56.4 ± 0.10
DAGs	8.8 ± 0.22	9.2 ± 0.03	13.2 ± 0.32	8.3 ± 0.25	8.8 ± 0.14	12.7 ± 0.28
MAGs	0.4 ± 0.03	0.6 ± 0.02	1.3 ± 0.06	0.5 ± 0.01	0.6 ± 0.13	1.1 ± 0.01
FFAs	8.2 ± 0.45	6.1 ± 0.06	30.7 ± 0.47	7.1 ± 0.08	5.6 ± 0.18	29.8 ± 0.17

Abbreviations: PO, crude palm oil; ROPO, refined olive pomace oil; OPAO, olive pomace acid oil; FAs, fatty acids; SFAs, saturated fatty acids (sum of C12:0, C14:0, C16:0, C18:0, C20:0, C22:0, and C24:0; see Table S2 in Supplementary Materials); MUFAs, monounsaturated fatty acids (sum of C16:1 n-7, C18:1 n-9, C18:1 n-7, and C20:1 n-9; see Table S2); PUFAs, polyunsaturated fatty acids (sum of C18:2 n-6, and C18:3 n-3); Ts, tocopherols; T3s, tocotrienols; T + T3, total tocol (sum of α-T, β-T, γ-T, δ -T, α-T3, β-T3, γ-T3, and δ-T3); TAGs, triacylglycerols; DAGs, diacylglycerols; MAGs, monoacylglycerols; FFAs, free fatty acids. ^1^ Data were expressed as mean ± standard deviation of two determinations. ^2^ The percentage of each FA was obtained by peak area normalization. See Table S2 for the complete FA composition, including minor FA results. ^3^ The percentage of each lipid class was obtained by peak area normalization.

**Table 4:** Fatty acid profile (%) of fresh dark chicken meat with skin coming from the three experimental diets.

FAs	PO ^1^	ROPO ^1^	OPAO ^1^	SEM	*p* ^2^
C16:0	26.0 ^a^	20.7 ^b^	21.4 ^b^	0.243	**<0.001**
C18:0	5.6	5.3	5.5	0.110	0.273
SFAs	32.7 ^a^	26.8 ^b^	27.7 ^b^	0.311	**<0.001**
C16:1 n-9	0.4 ^b^	0.5 ^a^	0.5 ^a^	0.014	**<0.001**
C16:1 n-7	3.8	3.4	3.5	0.120	0.097
C18:1 n-9	39.1 ^b^	44.8 ^a^	42.6 ^a^	0.592	**<0.001**
C18:1 n-7	2.0 ^b^	2.4 ^a^	2.3 ^a^	0.041	**<0.001**
MUFAs	45.7 ^b^	51.6 ^a^	49.4 ^a^	0.640	**<0.001**
C18:2 n-6	19.1	19.0	20.1	0.332	**0.046**
C20:4 n-6	0.7	0.7	0.8	0.027	0.170
n-6 PUFAs	20.5	20.4	21.6	0.364	**0.048**
C18:3 n-3	0.9 ^c^	1.0 ^b^	1.1 ^a^	0.013	**<0.001**
n-3 PUFAs	0.9 ^b^	1.1 ^a^	1.1 ^a^	0.014	**<0.001**
Total PUFAs	21.5	21.4	22.7	0.375	**0.039**

Abbreviations: PO, crude palm oil; ROPO, refined olive pomace oil; OPAO, olive pomace acid oil; FAs, fatty acids; SFAs, saturated fatty acids (sum of C12:0, C14:0, C15:0, C16:0, C17:0, and C18:0; see Table S3 in Supplementary Materials); MUFAs, monounsaturated fatty acids (sum of C14:1, C16:1 n-9, C16:1 n-7, C18:1 n-9, C18:1 n-7, and C20:1 n-9; see Table S3); PUFAs, polyunsaturated fatty acids (n-6 PUFAs: sum of C18:2 n-6, C18:3 n-6, C20:2 n-6, C20:3 n-6, and C20:4 n-6; n-3 PUFAs sum of C18:3 n-3, C20:5 n-3, and C22:6 n-3; Total PUFAs: sum of n-3 PUFAs and n-6 PUFAs; see Table S3); SEM, standard error of the mean. ^1^ Least-squares means of the different experimental units from each dietary treatment (n = 8). The percentage of each FA was obtained by peak area normalization. See Table S3 for the complete FA composition, including minor FA results. ^2^ *p* values obtained by ANOVA (n = 24). Values in bold were significant (*p* < 0.05). Differences between diets found with Scheffé’s post hoc test were noted in the same row as a > b > c. For C18:2 n-6, n-6 PUFAs, and total PUFAs, Scheffé’s post hoc test could not differentiate the least-squares means of the dietary treatments.

**Table 5:** Tocopherol and tocotrienol content (mg/kg) of fresh and refrigerated dark chicken meat with skin.

	α-T	γ-T	α-T3	T + T3
Effect of the diet on fresh chicken meat				
PO ^1^	11.19 ^a^	0.70	0.54 ^a^	12.68 ^a^
ROPO ^1^	10.37 ^ab^	0.78	0.07 ^b^	11.45 ^ab^
OPAO ^1^	9.59 ^b^	0.77	0.08 ^b^	10.74 ^b^
SEM	0.346	0.039	0.023	0.392
*p*_diet_ ^2^	**0.013**	0.319	**<0.001**	**0.007**
Effect of the diet on refrigerated chicken meat				
PO ^1^	10.89 ^a^	0.74 ^b^	0.41 ^a^	12.59 ^a^
ROPO ^1^	10.13 ^ab^	0.86 ^a^	0.01 ^b^	11.32 ^b^
OPAO ^1^	9.36 ^b^	0.83 ^ab^	0.03 ^b^	10.44 ^b^
SEM	0.231	0.029	0.038	0.313
*p*_diet_ ^2^	**0.001**	**0.028**	**<0.001**	**<0.001**
Effect of refrigeration on chicken meat				
Fresh chicken meat ^3^	10.38	0.75	0.23	11.62
Refrigerated chicken meat ^3^	10.13	0.81	0.15	11.45
SEM	0.170	0.020	0.018	0.205
*p*_refrigeration_ ^4^	0.289	**0.041**	**0.005**	0.556
Effect of the interaction between refrigeration and diet				
*p*_refrigeration × diet_ ^4^	0.992	0.833	0.362	0.951

Abbreviations: PO, crude palm oil; ROPO, refined olive pomace oil; OPAO, olive pomace acid oil; Ts, tocopherols; T3s, tocotrienols; T + T3, sum of α-T, β-T, γ-T, α-T3, β-T3, and γ-T3; SEM, standard error of the mean. ^1^ Least-squares means of the different experimental units for each dietary treatment (n = 8). ^2^ *p* values obtained from ANOVA (n = 24) of fresh or refrigerated chicken meat. Values in bold were significant (*p* < 0.05). Differences between diets found in fresh or refrigerated chicken meat with Scheffé’s post hoc test (n = 24) were noted in the same column as a > b. ^3^ Pooled means (least-squares means) of fresh or refrigerated chicken meat coming from the three dietary treatments (n = 24). ^4^ *p* values obtained for the refrigeration (*p*_refrigeration_) and the interaction between the refrigeration time and the diet (*p*_refrigeration × diet_) from multifactor ANOVA (n = 48). Values in bold were significant (*p* < 0.05).

**Table 6:** Volatile compounds (µg/kg) of fresh and refrigerated dark chicken meat with skin.

	Hexanal ^1^	2-Hexenal ^1^	Nonanal ^1^	1-Pentanol ^1^	1-Hexanol ^1^	1-Octen-3-ol ^1^	1-Octen-3-one ^1^	2-Pentylfuran ^1^
Effect of the diet on fresh chicken meat								
PO ^2^	5.7	4.5	1	1	2.6	11.1	0.8	6.1
ROPO ^2^	4.2	4.3	0.8	0.9	2.4	9.2	0.7	5.7
OPAO ^2^	4.6	3.9	0.7	1.1	3.3	14.3	0.7	5.5
SEM	0.782	0.533	0.073	0.116	0.305	1.438	0.068	0.525
*p*_diet_ ^3^	0.394	0.698	0.128	0.556	0.124	0.058	0.848	0.695
Effect of the diet on refrigerated chicken meat								
PO ^2^	10	6.7	0.9	2.7	4	24.5	0.8	5.7
ROPO ^2^	7.4	5.5	0.6	2.6	3.8	20.4	0.9	5.3
OPAO ^2^	6.5	4.3	0.5	2.3	3.2	17.8	0.7	4.5
SEM	1.407	0.85	0.168	0.237	0.454	2.965	0.077	0.671
*p*_diet_ ^3^	0.206	0.152	0.271	0.558	0.475	0.295	0.147	0.449
Effect of the refrigeration on chicken meat								
Fresh chicken meat ^4^	4.8	4.2	0.8	1	2.8	11.5	0.72	5.74
Refrigerated chicken meat ^4^	8	5.5	0.7	2.5	3.7	20.9	0.79	5.17
SEM	0.657	0.409	0.075	0.108	0.233	1.345	0.042	0.348
*p*_refrigeration_ ^5^	**0.002**	**0.038**	0.291	**<0.001**	**0.007**	**<0.001**	0.233	0.252
Effect of the interaction between refrigeration and diet								
*p*_refrigeration × diet_ ^5^	0.575	0.442	0.799	0.399	0.109	0.096	0.314	0.871

Abbreviations: PO, crude palm oil; ROPO, refined olive pomace oil; OPAO, olive pomace acid oil; SEM, standard error of the mean. ^1^ Volatile compound contents were expressed as 4-metil-2-pentanol equivalents. ^2^ Least-squares means of the different experimental units for each dietary treatment (n = 8). ^3^ *p* values obtained from ANOVA (n = 24) of fresh or refrigerated chicken meat. Values in bold were significant (*p* < 0.05). ^4^ Pooled means (least-squares means) of fresh or refrigerated chicken meat coming from the three dietary treatments (n = 24). ^5^ *p* values obtained for the refrigeration (*p*_refrigeration_) and the interaction between the refrigeration time and the diet (*p*_refrigeration × diet_) from multifactor ANOVA (n = 48). Values in bold were significant (*p* < 0.05).

**Table 7:** Color parameters (L*, a*, b* instrumental parameters, ΔE_R_, and ΔE_D_) and overall consumer acceptance of fresh and refrigerated dark chicken meat with skin.

	Color Parameters					Overall Acceptance
	L*	a*	b*	ΔE_R_ ^1^	ΔE_D_ ^1^	Sensory Scores
				(Fresh vs. Refrigerated)	(OPAO vs. Other Diets)	
Effect of the diet on fresh chicken meat						
PO ^2^	64.97	15.33	23.10	2.23	NC	5.94
ROPO ^2^	65.15	14.46	23.18	2.94	NC	6.44
OPAO ^2^	65.77	14.18	22.56	2.63		6.59
SEM	0.475	0.386	0.26			0.318
*p*_diet_ ^3^	0.466	0.115	0.215			0.318
Effect of the diet on refrigerated chicken meat						
PO ^2^	65.08 ^b^	17.53	23.60 ^b^		1.28	6.66
ROPO ^2^	66.67 ^a^	16.35	24.88 ^a^		1.72	6.53
OPAO ^2^	66.01 ^ab^	16.69	23.31 ^b^			6.56
SEM	0.343	0.332	0.22			0.244
*p*_diet_ ^3^	**0.012**	0.057	<0.001			0.931
Effect of the refrigeration on chicken meat						
Fresh chicken meat ^4^	65.3	14.66	22.95			6.32
Refrigerated chicken meat ^4^	65.92	16.86	23.93			6.58
SEM	0.239	0.208	0.139			0.164
*p*_refrigeration_ ^5^	0.071	**<0.001**	<0.001			0.262
Effect of the interaction between refrigeration and diet on chicken meat						
*p*_refrigeration × diet_ ^5^	0.181	0.697	0.04			0.368

Abbreviations: PO, crude palm oil; ROPO, refined olive pomace oil; OPAO, olive pomace acid oil; SEM, standard error of the mean; L*, lightness; a*, redness; b*, yellowness; NC, not calculated as there were no significant differences between diets. ^1^ ΔE_R_ values were calculated by Equation (1) and ΔE_D_ values were calculated by Equation (2).^2^ Least-squares means of the different experimental units for each dietary treatment (n = 8 for color parameters and n = 36 for sensory scores). ^3^ *p* values obtained from ANOVA (n = 24 for color parameters and n = 108 for sensory scores) of fresh or refrigerated chicken meat. Values in bold were significant (*p* < 0.05). Differences between diets found with Scheffé’s post hoc test were noted in the same row as a > b. ^4^ Pooled means (least-squares means) of fresh or refrigerated chicken meat coming from the three dietary treatments (n = 24 for color parameters and n = 108 for sensory scores). ^5^ *p* values obtained for the refrigeration (*p*_refrigeration_) and the interaction between the refrigeration time and the diet (*p*_refrigeration × diet_) obtained from multifactor ANOVA (n = 48 for color parameters and n = 216 for sensory scores). Values in bold were significant (*p* < 0.05).

## Data Availability

The data is contained within the article and Supplementary Materials to the article.

## Supplementary Materials

The following supporting information can be downloaded at: https://www.mdpi.com/article/10.3390/ani13081343/s1, Method for the determination of fatty acid composition of experimental diets; Method for the determination of tocopherol and tocotrienol content of experimental diets; Method for the determination of lipid class composition of experimental diets; Detailed descriptions of the following methods applied to dark chicken meat: determination of the fatty acid composition, determination of the tocopherol and tocotrienol content, ferrous oxidation-xylenol orange assay, determination of the 2-thiobarbituric acid value, and determination of the volatile compound content. Table S1. Complete fatty acid profile of the experimental fat sources; Table S2. Complete fatty acid profile of the experimental diets; Table S3. Complete fatty acid profile of fresh dark chicken meat with skin coming from the three experimental diets [54].

## References

[1] International Olive Council 2020/21 Crop Year: Production Down, Consumption Up. https://www.internationaloliveoil.org/2020-21-crop-year-production-down-consumption-up/.

[2] Khdair A., Abu-Rumman G. (2020). Sustainable Environmental Management and Valorization Options for Olive Mill Byproducts in the Middle East and North Africa (MENA) Region. Processes.

[3] Difonzo G., Troilo M., Squeo G., Pasqualone A., Caponio F. (2021). Functional Compounds from Olive Pomace to Obtain High-Added Value Foods—A Review. J. Sci. Food Agric..

[4] Varona E., Tres A., Rafecas M., Vichi S., Barroeta A.C., Guardiola F. (2021). Composition and Nutritional Value of Acid Oils and Fatty Acid Distillates Used in Animal Feeding. Animals.

[5] Verge-Mèrida G., Solà-Oriol D., Tres A., Verdú M., Farré G., Garcés-Narro C., Barroeta A.C. (2022). Olive Pomace Oil and Acid Oil as Alternative Fat Sources in Growing-Finishing Broiler Chicken Diets. Poult. Sci..

[6] Verge-Mèrida G., Barroeta A.C., Ferrer C., Serrano T., Guardiola F., Soler M.D., Sala R. (2022). Olive Pomace and Soybean-Sunflower Acid Oils as Alternative Fat Sources in European Seabass (*Dicentrarchus labrax*) Diets: Effects on Performance, Digestibility and Flesh Fatty Acid Composition and Quality Parameters. Animals.

[7] Verge-Mèrida G., Barroeta A.C., Guardiola F., Verdú M., Balart M., Font-i-Furnols M., Solà-Oriol D. (2021). Crude and Acid Oils from Olive Pomace as Alternative Fat Sources in Growing-Finishing Pigs. Animal.

[8] Albendea P., Tres A., Rafecas M., Vichi S., Sala R., Guardiola F. (2023). Effect of Feeding Acid Oils on European Seabass Fillet Lipid Composition, Oxidative Stability, Color, and Sensory Acceptance. Aquac. Nutr..

[9] Varona E., Tres A., Rafecas M., Vichi S., Barroeta A.C., Guardiola F. (2021). Methods to Determine the Quality of Acid Oils and Fatty Acid Distillates Used in Animal Feeding. MethodsX.

[10] European Commission (2010). EU Directive 2010/63/EU of the European Parliament and of the Council of 22 September 2010 on the Protection of Animals Used for Scientific Purposes. Off. J. Eur. Union..

[11] Santomá G., Mateos G.G., FEDNA (2018). Necesidades Nutricionales en Avicultura.

[12] Bou R., Guardiola F., Tres A., Barroeta A.C., Codony R. (2004). Effect of Dietary Fish Oil, α-Tocopheryl Acetate, and Zinc Supplementation on the Composition and Consumer Acceptability of Chicken Meat. Poult. Sci..

[13] Aleman M., Nuchi C.D., Bou R., Tres A., Polo J., Guardiola F., Codony R. (2010). Effectiveness of Antioxidants in Preventing Oxidation of Palm Oil Enriched with Heme Iron: A Model for Iron Fortification in Baked Products. Eur. J. Lipid Sci. Technol..

[14] Grau A., Codony R., Rafecas M., Barroeta A.G., Guardiola F. (2000). Lipid Hydroperoxide Determination in Dark Chicken Meat through a Ferrous Oxidation-Xylenol Orange Method. J. Agric. Food Chem..

[15] Tres A., Nuchi C.D., Bou R., Codony R., Guardiola F. (2009). Assessing Rabbit and Chicken Tissue Susceptibility to Oxidation through the Ferrous Oxidation-Xylenol Orange Method. Eur. J. Lipid Sci. Technol..

[16] Grau A., Guardiola F., Boatella J., Barroeta A., Codony R. (2000). Measurement of 2-Thiobarbituric Acid Values in Dark Chicken Meat through Derivative Spectrophotometry: Influence of Various Parameters. J. Agric. Food Chem..

[17] Botsoglou N.A., Fletouris D.J., Papageorgiou G.E., Vassilopoulos V.N., Mantis A.J., Trakatellis A.G. (1994). Rapid, Sensitive, and Specific Thiobarbituric Acid Method for Measuring Lipid Peroxidation in Animal Tissue, Food, and Feedstuff Samples. J. Agric. Food Chem..

[18] FEDNA Normas FEDNA de Control de Calidad de Ingredientes para Piensos-Especificación Técnica de Materia Prima, Grasas y Aceites. www.fundacionfedna.org/sites/%0Adefault/files/8_Grasas_y_aceites.pdf.

[19] Varona E., Tres A., Rafecas M., Vichi S., Sala R., Guardiola F. (2021). Oxidative Quality of Acid Oils and Fatty Acid Distillates Used in Animal Feeding. Animals.

[20] Cort W.M., Vicente T.S., Waysek E.H., Williams B.D. (1983). Vitamin E Content of Feedstuffs Determined by High-Performance Liquid Chromatographic Fluorescence. J. Agric. Food Chem..

[21] Ai Y., Jane J.L. (2016). Macronutrients in Corn and Human Nutrition. Compr. Rev. Food Sci. Food Saf..

[22] Kan A. (2015). Characterization of the Fatty Acid and Mineral Compositions of Selected Cereal Cultivars from Turkey. Rec. Nat. Prod..

[23] de Oliveira C.O., Roll A.A.P., Medeiros Gonçalves F.M., Lopes D.C.N., Xavier E.G. (2021). Olive Pomace for the Feeding of Commercial Poultry: Effects on Performance, Meat and Eggs Quality, Haematological Parameters, Microbiota and Immunity. World’s Poult. Sci. J..

[24] Wood J.D., Enser M., Purslow P.P. (2017). Manipulating the Fatty Acid Composition of Meat to Improve Nutritional Value and Meat Quality. New Aspects of Meat Quality. From Genes to Ethics.

[25] Grashorn M.A. (2007). Functionality of Poultry Meat. J. Appl. Poult. Res..

[26] Bou R., Codony R., Tres A., Decker E.A., Guardiola F. (2009). Dietary Strategies to Improve Nutritional Value, Oxidative Stability, and Sensory Properties of Poultry Products. Crit. Rev. Food Sci. Nutr..

[27] Zaki E.F., El Faham A.I., Nematallah G.M. (2018). Fatty Acids Profile and Quality Characteristics of Broiler Chicken Meat Fed Different Dietary Oil Sources with Some Additives. Int. J. Health Anim. Sci. Food Saf..

[28] Crespo N., Esteve-Garcia E. (2001). Dietary Fatty Acid Profile Modifies Abdominal Fat Deposition in Broiler Chickens. Poult. Sci..

[29] O’Neill L.M., Galvin K., Morrissey P.A., Buckley D.J. (1998). Comparison of Effects of Dietary Olive Oil, Tallow and Vitamin E on the Quality of Broiler Meat and Meat Products. Br. Poult. Sci..

[30] Kang K.R., Cherian G., Sim J.S. (1998). Tocopherols, Retinol and Carotenes in Chicken Egg and Tissues as Influenced by Dietary Palm Oil. J. Food Sci..

[31] Qi J., Wang H.H., Zhou G.H., Xu X.L., Li X., Bai Y., Yu X. (2017). Evaluation of the Taste-Active and Volatile Compounds in Stewed Meat from the Chinese Yellow-Feather Chicken Breed. Int. J. Food Prop..

[32] Addeen A., Benjakul S., Maqsood S. (2016). Haemoglobin-Mediated Lipid Oxidation in Washed Chicken Mince. Indian J. Sci. Technol..

[33] Ercolini D., Russo F., Nasi A., Ferranti P., Villani F. (2009). Mesophilic and Psychrotrophic Bacteria from Meat and Their Spoilage Potential in Vitro and in Beef. Appl. Environ. Microbiol..

[34] Casaburi A., Piombino P., Nychas G.J., Villani F., Ercolini D. (2015). Bacterial Populations and the Volatilome Associated to Meat Spoilage. Food Microbiol..

[35] Zhang C., Zhang H., Liu M., Zhao X., Luo H. (2020). Effect of Breed on the Volatile Compound Precursors and Odor Profile Attributes of Lamb Meat. Foods.

[36] Qi J., Liu D.Y., Zhou G.H., Xu X.L. (2017). Characteristic Flavor of Traditional Soup Made by Stewing Chinese Yellow-Feather Chickens. J. Food Sci..

[37] Chen D.W., Balagiannis D.P., Parker J.K. (2019). Use of Egg Yolk Phospholipids to Generate Chicken Meat Odorants. Food Chem..

[38] Botsoglou N.A., Christaki E., Fletouris D.J., Florou-Paneri P., Spais A.B. (2002). The Effect of Dietary Oregano Essential Oil on Lipid Oxidation in Raw and Cooked Chicken during Refrigerated Storage. Meat Sci..

[39] Ryu Y.C., Rhee M.S., Lee M.H., Lee S.K., Kim B.C. (2006). Effects of Packaging Methods on the Meat Quality of α-Tocopherol Supplemented Broiler Chicks during Refrigerated Storage. Food Sci. Biotechnol..

[40] Tománková J., Borilová G., Steinhauserová I., Las L.G. (2012). Volatile Organic Compounds as Biomarkers of the Freshness of Poultry Meat Packaged in a Modified Atmosphere. Czech J. Food Sci..

[41] Arvanitoyannis I.S., Stratakos A.C. (2012). Application of Modified Atmosphere Packaging and Active/Smart Technologies to Red Meat and Poultry: A Review. Food Bioprocess Technol..

[42] Narciso-Gaytán C., Shin D., Sams A.R., Bailey C.A., Miller R.K., Smith S.B., Leyva-Ovalle O.R., Sánchez-Plata M.X. (2010). Soybean, Palm Kernel, and Animal-Vegetable Oils and Vitamin E Supplementation Effect on Lipid Oxidation Stability of Sous Vide Chicken Meat. Poult. Sci..

[43] Grau A., Guardiola F., Grimpa S., Barroeta A.C., Codony R. (2001). Oxidative Stability of Dark Chicken Meat through Frozen Storage: Influence of Dietary Fat and α-Tocopherol and Ascorbic Acid Supplementation. Poult. Sci..

[44] Lauridsen C., Buckley D.J., Morrissey P.A. (1997). Influence of Dietary Fat and Vitamin E Supplementation on α-Tocopherol Levels and Fatty Acid Profiles in Chicken Muscle Membranal Fractions and on Susceptibility to Lipid Peroxidation. Meat Sci..

[45] Lebret B., Čandek-Potokar M. (2022). Review: Pork Quality Attributes from Farm to Fork. Part I. Carcass and Fresh Meat. Animal.

[46] Zhang H., Wu J., Guo X. (2016). Effects of Antimicrobial and Antioxidant Activities of Spice Extracts on Raw Chicken Meat Quality. Food Sci. Hum. Wellness.

[47] Faustman C., Sun Q., Mancini R., Suman S.P. (2010). Myoglobin and Lipid Oxidation Interactions: Mechanistic Bases and Control. Meat Sci..

[48] Pogorzelska E., Godziszewska J., Brodowska M., Wierzbicka A. (2018). Antioxidant Potential of Haematococcus Pluvialis Extract Rich in Astaxanthin on Colour and Oxidative Stability of Raw Ground Pork Meat during Refrigerated Storage. Meat Sci..

[49] Chiu M.C., de Morais Coutinho C., Gonçalves L.A.G. (2009). Carotenoids Concentration of Palm Oil Using Membrane Technology. Desalination.

[50] Criado M.N., Romero M.P., Motilva M.J. (2007). Effect of the Technological and Agronomical Factors on Pigment Transfer during Olive Oil Extraction. J. Agric. Food Chem..

[51] Boskou D., Gunstone F.D. (2011). Olive Oil. Vegetable Oils in Food Technology: Composition, Properties and Uses.

[52] Grashorn M., Carle R., Schweiggert R.M. (2016). Feed Additives for Influencing Chicken Meat and Egg Yolk Color. Handbook on Natural Pigments in Food and Beverages: Industrial Applications for Improving Food Color.

[53] Gray J.I., Gomaa E.A., Buckley D.J. (1996). Oxidative Quality and Shelf Life of Meats. Meat Sci..

[54] AOAC International (2019). Official Methods of Analysis of AOAC International.

