# Supplementary material for: Effect of Feeding Olive Pomace Acid Oil on Dark Chicken Meat Lipid Composition, Oxidative Stability, Color, and Sensory Acceptance
Source: Animals (Basel). 2023 Apr 13;13(8):1343. doi: 10.3390/ani13081343 (PMC10134984; doi:10.3390/ani13081343)
Supplement: Supplementary file 1 [file animals-13-01343-s001.zip › Supplementary v3.docx]

Effect of feeding olive pomace acid oil on dark chicken meat lipid composition, oxidative stability, color, and sensory acceptance – Supplementary Materials

Paula Albendea ^1,2^, Francesc Guardiola ^1,2^*, Magdalena Rafecas ^2,3^, Stefania Vichi ^1,2^, Ana C. Barroeta ^4^, Marçal Verdú ^5^ and Alba Tres^1,2,^

^1^ Nutrition, Food Science and Gastronomy Departament-XIA, Facultat de Farmàcia i Ciències de l’Alimentació, Campus de l’Alimentació de Torribera, Universitat de Barcelona, Av. Prat de la Riba, E-08921 Santa Coloma de Gramenet, Spain; paula.albendea4@ub.edu; stefaniavichi@ub.edu; atres@ub.edu

^2^ Institut de Recerca en Nutrició i Seguretat Alimentària (INSA-UB), Universitat de Barcelona, Av. Prat de la Riba, E-08921 Santa Coloma de Gramenet, Spain

^3^ Nutrition, Food Science and Gastronomy Departament-XIA, Facultat de Farmàcia i Ciències de l’Alimentació, Campus Diagonal, Universitat de Barcelona, Av. de Joan XXIII, E-08028 Barcelona, Spain; magdarafecas@ub.edu

^4^ Animal Nutrition and Welfare Service (SNiBA), Animal and Food Science Department, Facultat de Veterinària, Universitat Autònoma de Barcelona, Edifici V, Travessera dels Turons, E-08193 Bellaterra, Spain; ana.barroeta@uab.cat

^5^ Department of Animal Nutrition and Feed Industry, bonÀrea Agrupa, E-25210 Guissona, Spain; marsal.verdu@bonarea.com

***** Correspondence: [fguardiola@ub.edu](mailto:fguardiola@ub.edu); Tel.: +34-934-03-71-96

*Animals, 2023, XXX.*

Method for the determination of fatty acid composition of experimental diets

Lipids were extracted in duplicate from 2 g of feed by using diethyl ether according to the Soxtec extraction method 2003.05 of AOAC International [1]. After extraction, the total evaporation of the solvent was avoided to prevent oxidation and more diethyl ether was added to transfer the lipid extract from Soxtec vessels to two glass test tubes with screw cap (approximately half of the lipid extract to each tube). Diethyl ether was completely evaporated from tubes under N_2_ stream at 30 °C in a block heater and the lipid extract was stored at −20 °C until the analyses. One tube was used for the determination of the fatty acid (FA) composition and the other to analyze the lipid class composition, as described below. For FA determination, the lipid extract was submitted to a double methylation in methanolic medium and FA methyl esters (FAMEs) were determined by gas chromatography with flame ionization detector (GC-FID) [2]. Each compound was identified by comparing its retention time with that of standards (Supelco 37 component FAME Mix, Supelco®, Merck KGaA, Darmstadt, Germany) and the percentage of each FA was obtained by peak area normalization.

Method for the determination of tocopherol and tocotrienol content of experimental diets

The tocopherol and tocotrienol was determined in duplicate as described by Bou et al. [3]. Briefly, 1.5 g of feed was homogenized with a mixture of antioxidants in ethanol using a high-speed homogenizer (PT 3100 Polytron, Kinematica, Lucerne, Switzerland) for 30 s at 20,000 rpm and saponified with methanolic KOH. After saponification, the non-saponifiable fraction was extracted with petroleum ether and filtered through a 0.45-μm Teflon membrane. After complete evaporation of the petroleum ether under a nitrogen stream at 30 °C in a block heater, the residue was redissolved in an exact volume of 99% n-hexane and injected into the high-performance liquid chromatograph (HPLC) system. HPLC was performed as explained by Aleman et al. [4] and a 1260 Infinity II Fluorescence Detector (Agilent Technologies, Santa Clara, CA, USA) was used with the excitation and emission wavelengths settled to 290 and 320 nm, respectively. Calibration curves were prepared for each tocopherol, using a set of standards (α-, β-, γ- and δ-tocopherol) from Calbiochem® (Merck KGaA, Darmstadt, Germany). Each tocotrienol (α-, β-, γ- and δ-tocotrienol) was quantified using the corresponding tocopherol calibration curve.

Method for the determination of lipid class composition of experimental diets

The lipid extraction of the feeds by the Soxtec method was performed as described above in duplicate. The lipid extract was dissolved in 2 mL of tetrahydrofuran (HPLC grade from Scharlau, Sentmenat, Spain) and a 1:2 dilution was made to obtain a lipid concentration of ≈ 15 mg/mL. Then, the percentages of triacyclglycerols, diacylglycerols, monoacylglycerols and free fatty acids were determined by size molecular exclusion chromatography as described by Varona et al. [2] and quantified by peak area normalization.

Method for the determination of fatty acid composition of dark chicken meat

The fatty acid composition of fresh dark chicken meat was analyzed in duplicate by GC-FID after extracting the lipid fraction of 1g of sample with chloroform/methanol (2:1, v/v) mixture. To perform the extraction, first, 20mL of this solvent mixture was added to the sample and homogenized using a PT 3100 Polytron at 19,000 rpm for 20 s and filtered through Whatman No. 1 filter paper. The sample residue retained in the filter was reextracted twice with 10 mL of the same solvent mixture at 19,000 rpm for 20 s. Next steps of lipid extraction were carried out as described by Bou et al. [3]. Then, FAMEs were obtained from the extracted lipid fraction by a double methylation procedure and determined by GC-FID [2]. Each compound was identified by comparing its retention time with that of standards (Supelco 37 component FAME Mix, Supelco®, Merck KGaA, Darmstadt, Germany). The percentage of each FA was obtained by peak area normalization.

Method for the determination of tocopherol and tocotrienol content of dark chicken meat

For the determination of the tocopherol and tocotrienol content in fresh and refrigerated chicken meat, 2 g of sample was homogenized with a mixture of antioxidants in ethanol using a PT 3100 Polytron for 30 s at 20,000 rpm and saponified with methanolic KOH as described by Bou et al. [3]. The nonsaponifiable fraction was extracted with petroleum ether and filtered through a 0.45-μm Teflon membrane. The solvent was completely evaporated under a nitrogen stream at 30°C in a block heater and the residue was redissolved in an exact volume of 99% n-hexane and injected into the HPLC system. HPLC separation was carried out as described by Aleman et al. [4] and tocopherols and tocotrienols were detected using a 1260 Infinity II Fluorescence Detector (Agilent Technologies, Santa Clara, CA, USA), setting the excitation and emission wavelengths at 290 and 320nm, respectively. Calibration curves were prepared for each tocopherol, using a set of standards (α-, β-, γ- and δ-tocopherol) from Calbiochem® (Merck KGaA, Darmstadt, Germany). Each tocotrienol (α-, β-, γ- and δ-tocotrienol) was quantified using the corresponding tocopherol calibration curve.

Ferrous oxidation-xylenol orange assay

Ferrous oxidation-xylenol orange method was applied to each sample per triplicate to evaluate the primary oxidation and the oxidative stability of fresh and refrigerated dark chicken meat samples, as detailed by Grau et al.[5]. Briefly, 15 mL of cold methanol was added to 2 g of sample, and a PT 3100 Polytron was used for sample homogenization at 12,000 rpm for 30 s. Then, the mixture was centrifuged (1,400 g, 3 min), and the supernatant methanol extract was collected. The reaction mixture was prepared in glass cuvettes, capped with Teflon caps. The reagents were added as described by Tres et al.[6], using 940 µL of methanol and 160 µL of sample extract for fresh samples, and with 950 µL of methanol and 150 µL of sample extract for refrigerated samples. The absorbance at 560nm was measured using a UV-3600 spectrophotometer (Shimadzu, Kyoto, Japan) after incubation for 30 min (as a measure of the lipid hydroperoxides present in the samples from the beginning, named lipid hydroperoxide content) and for 96h (as a measure of the amount of lipid hydroperoxides formed during this time, which is considered a measure of the oxidative stability of the samples, named final lipid hydroperoxide value). Lipid hydroperoxide concentration was expressed as mmol of cumene hydroperoxide equivalents/kg of sample, with reference to a calibration curve prepared using cumene hydroperoxide as standard (technical grade, 80%, Sigma-Aldrich®, Merck KGaA, Darmstadt, Germany).

Method for the determination of 2-thiobarbituric acid value of dark chicken meat

The 2-thiobarbituric acid value was determined in duplicate to evaluate the secondary oxidation of fresh and refrigerated chicken meat, applying the method described by Grau et al. [7] on 1.5 g of sample. Briefly, the 2-thiobarbituric acid value was measured through third derivative spectrophotometry after an acid aqueous extraction. The results were expressed as malondialdehyde concentration (μg/kg), using a calibration curve prepared as described by Botsoglou et al. [8] with 1,1,3,3-tetraethoxypropane (96%, Sigma-Aldrich®, Merck KGaA, Darmstadt, Germany) as MDA precursor.

Method for the determination of volatile compound content of dark chicken meat

The volatile compound content was determined in fresh and refrigerated chicken meat by headspace solid-phase microextraction coupled with gas chromatography and mass spectrometry (HS-SPME-GC-MS) as described by Albendea et al. [9]. To perform the analysis, 1 g of sample was weighed into a 10mL screw-capped vial, and 0.5mL of a 4mg/L aqueous solution of 4-methyl-2-pentanol (97%, Sigma-Aldrich®, Merck KGaA, Darmstadt, Germany) was added as internal standard. Subsequently, 0.5mL of an aqueous antioxidant solution with 4% of EDTA and 0.4% of propyl gallate (both from Sigma-Aldrich®, Merck KGaA, Darmstadt, Germany), 2mL of double deionized water, and three glass balls were added. The vial was immediately closed and kept in ice until all sample set was prepared. Then, the mixture was homogenized using an ultrasound bath at 4°C for 10min. Samples were kept in ice at the dark until the HS-SPME-GC-MS determination was carried out. The instrument consisted of an Agilent 6890N Network GC system with an Agilent 5975C Inert MSD quadrupole mass spectrometer (both from Agilent Technologies Santa Clara, CA, USA) and a PAL autosampler (CTC Analytics, Zwingen, Switzerland) configured to perform SPME. After 10 min of sample conditioning at the extraction temperature (45°C), the fiber of divinylbenzene/carboxen/polydimethylsiloxane (2 cm length, 50/30 thickness) from Supelco® (Merck KGaA, Darmstadt, Germany) was exposed to the head space for 30 min and desorbed in the injector at 260°C for 10 min. To perform the separation of the different volatile compounds, a Supelcowax-10 capillary column (30m × 0:25mm i.d., 0.25 μm film thickness) from Supelco® (Merck KGaA, Darmstadt, Germany) was used. The oven temperature program began at 40°C (held 10 min, during fiber desorption time), 3°C/min up to 150°C, and 15°C/min up to 250°C (held for 5 min). Helium was used as gas carrier with a constant flow of 1mL/min. The temperatures of the ion source and the transfer line were 230 and 280°C, respectively, and the ionization energy was 70 eV. Data were acquired in full scan mode in selected representative samples for the identification of compounds, which was carried out by comparison of their mass spectra and retention times with those of standard compounds or with those available in mass spectrum library Wiley 6 and in the literature. Then, the quantitative assessment of all samples was carried by selected ion mode, considering m/z 44, 45, 55, 56, 57, 81, and 98, which were representative for the compounds of interest. Data were then analyzed by an Agilent MSD ChemStation. Relative amounts of volatile compounds were calculated by the internal standard method, expressing the results as μg of 4-methyl-2-pentanol equivalents/kg of sample.

Table S1. Complete fatty acid profile (%) of the experimental fat sources.

| **FAs** | **PO ^1^** | **ROPO ^1^** | **OPAO ^1^** |
| --- | --- | --- | --- |
| C14:0 | 1.0 ± 0.01 | ND | 0.1 ± 0.01 |
| C16:0 | 42.5 ± 0.05 | 12.7 ± 0.01 | 12.4 ± 0.14 |
| C18:0 | 4.5 ± 0.01 | 2.7 ± 0.01 | 2.7 ± 0.89 |
| C20:0 | 0.4 ± 0.01 | 0.5 ± 0.01 | 0.5 ± 0.01 |
| C22:0 | ND | 0.3 ± 0.01 | 0.5 ± 0.01 |
| C24:0 | ND | 0.1 ± 0.01 | 0.4 ± 0.01 |
| SFAs | 48.5 ± 0.03 | 16.2 ± 0.01 | 16.6 ± 0.74 |
| C16:1 n-7 | 0.2 ± 0.01 | 0.9 ± 0.01 | 1.0 ± 0.01 |
| C18:1 n-9 | 40.6 ± 0.05 | 70.0 ± 0.01 | 63.2 ± 0.61 |
| C18:1 n-7 | 0.6 ± 0.01 | 1.6 ± 0.01 | 1.7 ± 0.01 |
| C20:1 n-9 | 0.1 ± 0.01 | 0.3 ± 0.01 | 0.4 ± 0.01 |
| MUFAs | 41.6 ± 0.05 | 72.9 ± 0.02 | 66.2 ± 0.63 |
| C18:2 n-6 | 9.5 ± 0.01 | 10.1 ± 0.02 | 15.8 ± 0.12 |
| C18:3 n-3 | 0.3 ± 0.01 | 0.7 ± 0.01 | 0.9 ± 0.01 |
| PUFAs | 9.8 ± 0.01 | 10.8 ± 0.02 | 16.7 ± 0.13 |
| *trans-C18:1* | 0.1 ± 0.01 | 0.1 ± 0.01 | 0.4 ± 0.01 |

Abbreviations: PO, palm oil; ROPO, refined olive pomace oil; OPAO, olive pomace acid oil; FAs, fatty acids; SFAs, saturated fatty acids (sum of C14:0, C16:0, C18:0, C20:0, C22:0 and C24:0); MUFAs, monounsaturated fatty acids (sum of C16:1 n-7, C18:1 n-9, C18:1 n-7 and C20:1 n-9); PUFAs, polyunsaturated fatty acids (sum of C18:2 n-6 and C18:3 n-3); *trans*-C18:1 isomers (sum of positional isomers); ND, not detected. ^1^ Data were expressed as mean ± standard deviation of three determinations. The percentage of each FA was obtained by peak area normalization.

Table S2. Complete fatty acid profile (%) of the experimental diets.

|  | **Grower diets** | | |  | **Finisher diets** | | |
| --- | --- | --- | --- | --- | --- | --- | --- |
| FAs | PO ^1^ | ROPO ^1^ | OPAO ^1^ |  | PO ^1^ | ROPO ^1^ | OPAO ^1^ |
| C12:0 | 0.5 ± 0.01 | 0.1 ± 0.01 | 0.2 ± 0.01 |  | 0.3 ± 0.01 | ND | 0.1 ± 0.01 |
| C14:0 | 0.8 ± 0.01 | 0.1 ± 0.01 | 0.2 ± 0.01 |  | 0.8 ± 0.01 | 0.12 ± 0.01 | 0.3 ± 0.01 |
| C16:0 | 31.7 ± 0.04 | 13.0 ± 0.04 | 14.9 ± 0.02 |  | 32.6 ± 0.20 | 13.6 ± 0.01 | 16.5 ± 0.65 |
| C18:0 | 3.8 ± 0.01 | 2.7 ± 0.01 | 3.2 ± 0.01 |  | 3.8 ± 0.07 | 2.9 ± 0.01 | 3.2 ± 0.07 |
| C20:0 | 0.4 ± 0.01 | 0.4 ± 0.01 | 0.5 ± 0.01 |  | 0.4 ± 0.01 | 0.5 ± 0.01 | 0.5 ± 0.01 |
| C22:0 | 0.2 ± 0.01 | 0.3 ± 0.01 | 0.4 ± 0.01 |  | 0.2 ± 0.01 | 0.3 ± 0.01 | 0.4 ± 0.02 |
| C24:0 | 0.2 ± 0.01 | 0.2 ± 0.01 | 0.4 ± 0.01 |  | 0.2 ± 0.01 | 0.2 ± 0.01 | 0.4 ± 0.03 |
| SFAs | 37.6 ± 0.05 | 16.9 ± 0.05 | 19.7 ± 0.02 |  | 38.3 ± 0.12 | 17.6 ± 0.01 | 21.2 ± 0.81 |
| C16:1 n-7 | 0.2 ± 0.01 | 0.6 ± 0.01 | 0.6 ± 0.01 |  | 0.2 ± 0.01 | 0.1 ± 0.01 | 0.6 ± 0.02 |
| C18:1 n-9 | 35.5 ± 0.04 | 54.7 ± 0.08 | 46.7 ± 0.11 |  | 35.9 ± 0.33 | 55.0 ± 0.01 | 47.8 ± 1.41 |
| C18:1 n-7 | 1.1 ± 0.01 | 1.9 ± 0.03 | 1.7 ± 0.06 |  | 1.1 ± 0.02 | 1.8 ± 0.11 | 1.6 ± 0.08 |
| C20:1 n-9 | 0.2 ± 0.01 | 0.3 ± 0.01 | 0.3 ± 0.01 |  | 0.2 ± 0.01 | 0.3 ± 0.01 | 0.3 ± 0.01 |
| MUFAs | 36.9 ± 0.05 | 57.5 ± 0.04 | 49.3 ± 0.05 |  | 37.3 ± 0.32 | 57.3 ± 0.11 | 50.2 ± 1.51 |
| C18:2 n-6 | 24.6 ± 0.02 | 24.4 ± 0.06 | 29.6 ± 0.03 |  | 23.6 ± 0.19 | 24.0 ± 0.10 | 27.3 ± 0.69 |
| C18:3 n-3 | 0.9 ± 0.02 | 1.1 ± 0.02 | 1.3 ± 0.01 |  | 0.8 ± 0.01 | 1.0 ± 0.01 | 1.1 ± 0.01 |
| PUFAs | 25.5 ± 0.01 | 25.5 ± 0.09 | 30.8 ± 0.01 |  | 24.4 ± 0.20 | 25.0 ± 0.11 | 28.4 ± 0.70 |
| *trans*-C18:1 | ND | 0.1 ± 0.01 | 0.2 ± 0.02 |  | ND | 0.1 ± 0.01 | 0.2 ± 0.01 |

Abbreviations: PO, palm oil; ROPO, refined olive pomace oil; OPAO, olive pomace acid oil; FAs, fatty acids; SFAs, saturated fatty acids (sum of C12:0, C14:0, C16:0, C18:0, C20:0 C22:0 and C24:0); MUFAs, monounsaturated fatty acids (sum of C16:1 n-7, C18:1 n-9, C18:1 n-7 and C20:1 n-9); PUFAs, polyunsaturated fatty acids (sum of C18:2 n-6 and C18:3 n-3); *trans*-C18:1 isomers (sum of positional isomers); ND, not detected. ^1^ Data were expressed as mean ± standard deviation of two determinations. The percentage of each FA was obtained by peak area normalization.

Table S3. Complete fatty acid profile (%) of fresh dark chicken meat with skin coming from the three experimental diets.

| **FAs** | **PO ^1^** | **ROPO ^1^** | **OPAO ^1^** | **SEM ^2^** | ***p* ^2^** |
| --- | --- | --- | --- | --- | --- |
| C12:0 | 0.16^a^ | 0.07^b^ | 0.08^b^ | 0.006 | **<0.001** |
| C14:0 | 0.71^a^ | 0.44^b^ | 0.48^b^ | 0.014 | **<0.001** |
| C15:0 | 0.10^a^ | 0.09^b^ | 0.09^ab^ | 0.002 | **0.006** |
| C16:0 | 26.02^a^ | 20.72^b^ | 21.42^b^ | 0.243 | **<0.001** |
| C17:0 | 0.15^b^ | 0.16^a^ | 0.17^a^ | 0.003 | **<0.001** |
| C18:0 | 5.56 | 5.31 | 5.47 | 0.110 | 0.273 |
| SFAs | 32.70^a^ | 26.78^b^ | 27.73^b^ | 0.311 | **<0.001** |
| C14:1 | 0.12^a^ | 0.08^b^ | 0.10^ab^ | 0.007 | **0.004** |
| C16:1 n-9 | 0.44^a^ | 0.54^b^ | 0.53^b^ | 0.014 | **<0.001** |
| C16:1 n-7 | 3.78 | 3.41 | 3.50 | 0.120 | 0.097 |
| C18:1 n-9 | 39.05^b^ | 44.79^a^ | 42.61^a^ | 0.592 | **<0.001** |
| C18:1 n-7 | 2.01^b^ | 2.41^a^ | 2.30^a^ | 0.041 | **<0.001** |
| C20:1 n-9 | 0.29^b^ | 0.33^a^ | 0.31^ab^ | 0.007 | **0.001** |
| MUFAs | 45.69^b^ | 51.56^a^ | 49.35^a^ | 0.640 | **<0.001** |
| C18:2 n-6 | 19.14 | 18.96 | 20.13 | 0.332 | **0.046** |
| C18:3 n-6 | 0.26^b^ | 0.28^a^ | 0.28^a^ | 0.005 | **0.008** |
| C20:2 n-6 | 0.16 | 0.16 | 0.17 | 0.007 | 0.550 |
| C20:3 n-6 | 0.19 | 0.19 | 0.21 | 0.005 | 0.181 |
| C20:4 n-6 | 0.71 | 0.71 | 0.78 | 0.027 | 0.170 |
| n-6 PUFAs | 20.51 | 20.36 | 21.61 | 0.364 | **0.048** |
| C18:3 n3 | 0.93^c^ | 1.02^b^ | 1.07^a^ | 0.013 | **<0.001** |
| C20:5 n3 | 0.03 | 0.03 | 0.03 | 0.002 | 0.880 |
| C22:6 n3 | 0.04 | 0.05 | 0.05 | 0.002 | 0.316 |
| n-3 PUFAs | 0.96^b^ | 1.05^a^ | 1.10^a^ | 0.014 | **<0.001** |
| Total PUFAs | 21.47 | 21.40 | 22.71 | 0.377 | **0.039** |
| *trans*-C18:1 | 0.14^b^ | 0.25^a^ | 0.22^ab^ | 0.023 | **0.007** |

Abbreviations: PO, palm oil; ROPO, refined olive pomace oil; OPAO, olive pomace acid oil; FAs, fatty acids; SFAs, saturated fatty acids (sum of C12:0, C14:0, C15:0, C16:0, C17:0 and C18:0); MUFAs, monounsaturated fatty acids (sum of C14:1, C16:1 n-9, C16:1 n-7, C18:1 n-9, C18:1 n-7 and C20:1 n-9); PUFAs, polyunsaturated fatty acids (n-6 PUFAs: sum of C18:2 n-6, C18:3 n-6, C20:2 n-6, C20:3 n-6 and C20:4 n-6; n-3 PUFAs: sum of C18:3 n-3, C20:5 n-3 and C22:6 n-3; Total PUFAs: sum of n-3 PUFAs and n-6 PUFAs); trans-C18:1 isomers (sum of positional isomers); SEM, standard error of the mean. ^1^ Least-squares means of the different experimental units from each dietary treatment (n = 8). The percentage of each FA was obtained by peak area normalization. ^2^ *p* values obtained by ANOVA (n = 24). Values in bold were significant (*p* < 0.05). Differences between diets found with Scheffé’s post hoc test were noted in the same row as a > b > c. For C18:2 n-6, n-6 PUFAs and total PUFAs, Scheffé’s post hoc test could not differentiate the least-squares means of the dietary treatments.

References

1. AOAC International. *Official Methods of Analysis of AOAC International*; 21th ed.; AOAC International: Rockville, MD, USA, 2019; pp. 41-43.

2. Varona, E.; Tres, A.; Rafecas, M.; Vichi, S.; Barroeta, A.C.; Guardiola, F. Methods to determine the quality of acid oils and fatty acid distillates used in animal feeding. *MethodsX.* **2021**, *8*, 101334, doi:10.1016/j.mex.2021.101334.

3. Bou, R.; Guardiola, F.; Tres, A.; Barroeta, A.C.; Codony, R. Effect of dietary fish oil, α-tocopheryl acetate, and zinc supplementation on the composition and consumer acceptability of chicken meat. *Poult. Sci.* **2004**, *83*, 282–292, doi:10.1093/ps/83.2.282.

4. Aleman, M.; Nuchi, C.D.; Bou, R.; Tres, A.; Polo, J.; Guardiola, F.; Codony, R. Effectiveness of antioxidants in preventing oxidation of palm oil enriched with heme iron: A model for iron fortification in baked products. *Eur. J. Lipid Sci. Technol.* **2010**, *112*, 761–769, doi:10.1002/ejlt.200900220.

5. Grau, A.; Codony, R.; Rafecas, M.; Barroeta, A.G.; Guardiola, F. Lipid hydroperoxide determination in dark chicken meat through a ferrous oxidation-xylenol orange method. *J. Agric. Food Chem.* **2000**, *48*, 4136–4143, doi:10.1021/jf991054z.

6. Tres, A.; Nuchi, C.D.; Bou, R.; Codony, R.; Guardiola, F. Assessing rabbit and chicken tissue susceptibility to oxidation through the ferrous oxidation-xylenol orange method. *Eur. J. Lipid Sci. Technol.* **2009**, *111*, 563–573, doi:10.1002/ejlt.200800230.

7. Grau, A.; Guardiola, F.; Boatella, J.; Barroeta, A.; Codony, R. Measurement of 2-thiobarbituric acid values in dark chicken meat through derivative spectrophotometry: Influence of various parameters. *J. Agric. Food Chem.* **2000**, *48*, 1155–1159, doi:10.1021/jf990518q.

8. Botsoglou, N.A.; Fletouris, D.J.; Papageorgiou, G.E.; Vassilopoulos, V.N.; Mantis, A.J.; Trakatellis, A.G. Rapid, sensitive, and specific thiobarbituric acid method for measuring lipid peroxidation in animal tissue, food, and feedstuff samples. *J. Agric. Food Chem.* **1994**, *42*, 1931–1937, doi:10.1021/jf00045a019.

9. Albendea, P.; Tres, A.; Rafecas, M.; Vichi, S.; Sala, R.; Guardiola, F. Effect of feeding acid oils on European seabass fillet lipid composition, oxidative stability, color, and sensory acceptance. *Aquac. Nutr.* **2023**, *2023*, 6415693, doi:10.1155/2023/6415693.
